# Ecoregion Prioritization Suggests an Armoury Not a Silver Bullet for Conservation Planning

**DOI:** 10.1371/journal.pone.0008923

**Published:** 2010-01-27

**Authors:** Stephan M. Funk, Julia E. Fa

**Affiliations:** Durrell Wildlife Conservation Trust, Les Augrès Manor, Trinity, Jersey; University of Otago, New Zealand

## Abstract

In the face of accelerating species extinctions, map-based prioritization systems are increasingly useful to decide where to pursue conservation action most effectively. However, a number of seemingly inconsistent schemes have emerged, mostly focussing on endemism. Here we use global vertebrate distributions in terrestrial ecoregions to evaluate how continuous and categorical ranking schemes target and accumulate endangered taxa within the IUCN Red List, Alliance for Zero Extinction (AZE), and EDGE of Existence programme. We employed total, endemic and threatened species richness and an estimator for richness-adjusted endemism as metrics in continuous prioritization, and WWF's Global200 and Conservation International's (CI) Hotspots in categorical prioritization. Our results demonstrate that all metrics target endangerment more efficiently than by chance, but each selects unique sets of top-ranking ecoregions, which overlap only partially, and include different sets of threatened species. Using the top 100 ecoregions as defined by continuous prioritization metrics, we develop an inclusive map for global vertebrate conservation that incorporates important areas for endemism, richness, and threat. Finally, we assess human footprint and protection levels within these areas to reveal that endemism sites are more impacted but have more protection, in contrast to high richness and threat ones. Given such contrasts, major efforts to protect global biodiversity must involve complementary conservation approaches in areas of unique species as well as those with highest diversity and threat.

## Introduction

Biodiversity is vital for all humans. Despite a multitude of international agreements, local and global activism, academic debate and vast sums dedicated to conservation, the future of earth's natural capital remains uncertain [1]. Financial resources are highly limited and human pressure on land accelerating. Thus, ensuring the efficient allocation of resources for area selection, and thus maximum conservation impact, remains essential. Selection of “priority areas” for worldwide biodiversity conservation is a vital, but to a large extent unresolved exercise. Such areas aim to represent patterns and/or processes of biodiversity to be protected from threats to their persistence [2]. The selection of such areas is driven by the interpretation of underpinning biological data on species, habitats and biodiversity, and by threat assessments which may include socio-economic projections such as cost analysis, economics, likelihood of managing negative human interference and projections of anthropogenic induced threat [3], [4], [5], [6]. There has been a trend to seek a single set of priority areas for conservation, but divergent approaches and metrics have appeared that have resulted in an array of different projections. Most global prioritization focuses on concentrations of individual taxa or groups of species [7], predominantly centred on endemism [8], [9], [10]. All major institutional approaches to global biodiversity conservation prioritization operate on such an “irreplaceability/vulnerability” framework and aim for the protection of rare and endangered species rather than overall species diversity as the leading paradigm [7]. This may be because endemics have restricted distributions, often smaller populations, and thus greater vulnerability to extinction [7], [10], [11], whereas species richness is mainly driven by widespread and non-endangered organisms. Therefore, global conservation priorities based on richness alone have not been implemented [7], [10], [11] and are even regarded as of little practical use for conservation [11].

The metrics used to define biodiversity hotpots for conservation action remain highly controversial [12], [13]. The greatest challenges for priority-setting are the non-congruence of important areas defined by different indicator (surrogate) taxa and lack of correspondence of hotspots of species richness, endemism, and extinction threat [14], [15], [16], [17], [18], [19], [20], [21], [22]. Hotspots for sites of outstanding biodiversity in danger of accelerated destruction seldom coincide across taxa [17], [18], [13]. Similarly, high-resolution grid-based methods show a comparable distribution of species richness across vertebrate classes, but non-congruence for rare and threatened species and for surrogate taxa [20]. For terrestrial ecoregions [23], global patterns of richness and of endemism are highly correlated amongst four terrestrial vertebrate classes, whilst the correlation between richness and endemism is low [10]. Nevertheless, aggregate regions selected for high levels of endemism select more species than expected by chance alone, indicating that global distribution patterns of endemism can be used for the conservation of all terrestrial vertebrates [10]. The marked differences in cross-taxon congruence of endemic species emerging from the analysis of fine-scale grids [20] and ecoregions [10] might be scale-dependent [20], [24], but there is no *a priori* rationale for choice of optimal scale. Ecoregions are increasingly used as units for conservation, e.g. WWF's Global200 Ecoregions [25], because of their focus on natural units of distinct communities and species assemblages [23]. However, their full utility for priority setting remains unevaluated, especially the capture of threatened species by prioritization schemes other than endemism [10]. Here, we close this gap by investigating how prioritizing areas by species richness, endemism and threat target and accumulate different numbers of threatened taxa.

We test the performance and surrogacy of prioritized ecoregions in capturing threatened species. We use species richness, endemism, richness-adjusted endemism [26] (“∂-endemism”; see Methods), and IUCN Red List's threat categories [27] for 26,452 species of amphibians, reptiles, birds and mammals as selection metrics to rank 796 terrestrial ecoregions of the world [28]. The Red List aims to assess extinction risks but is not a prioritization system as it is confounded by taxonomic, ecological, geographical, political and socioeconomic variation between threatened taxa [29]. Nevertheless, the Red List is explicitly used for global priority setting, e.g. the Alliance for Zero Extinction, AZE, which focuses on species restricted to single sites and in danger of imminent extinction [30], and the EDGE of Existence programme, that jointly selects for evolutionary uniqueness and extinction risk [31]. We also evaluated the performance of the latest version of the Conservation International's (CI) Hotspots [32] and WWF's Global200 Ecoregions [25]. Both selection schemes identify but do not rank global priority areas and thus constitute categorical (i.e. binary) prioritization schemes. We then calculated the accumulation of threatened species using IUCN's “critically endangered”, CR, “endangered”, EN, AZE and EDGE of Existence programme as response measures (see Methods).

## Results and Discussion

**Figure 1:**
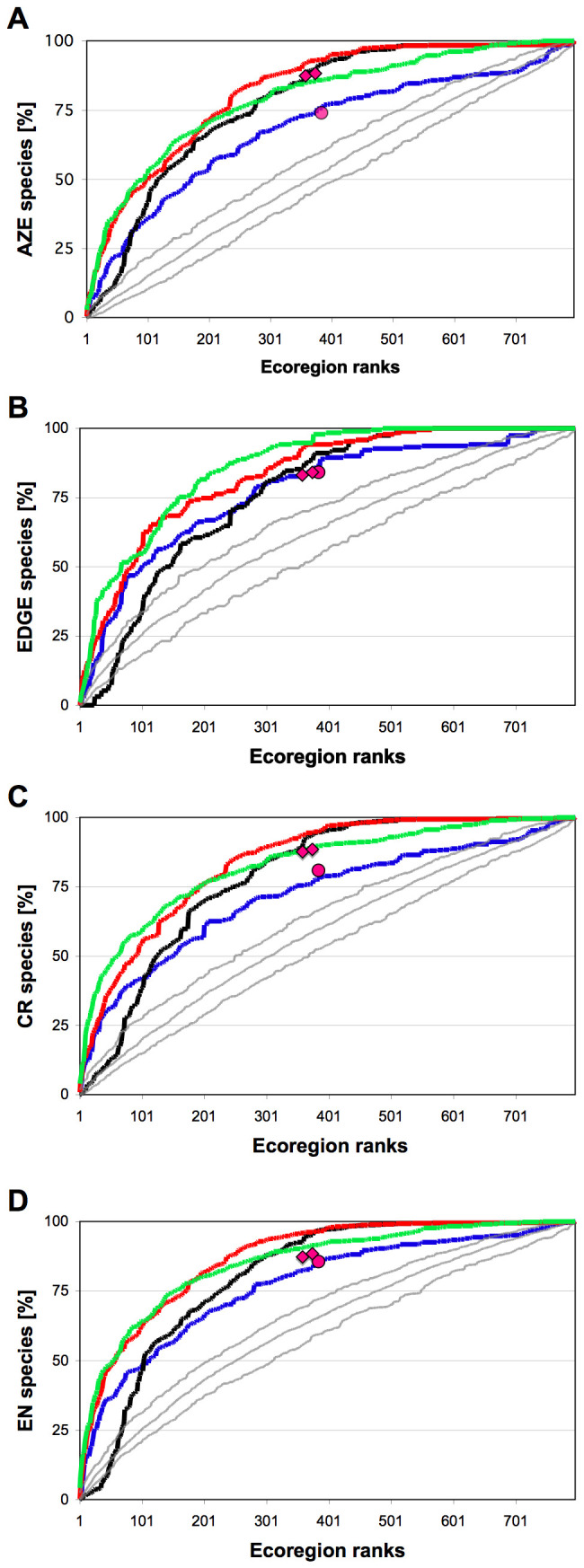
Accumulation of species over prioritized ecoregion ranks. Target species are EDGE (A), AZE (B), Red List's critically endangered CR (C) and Red List's endangered EN (D) species. Lines represent accumulations of species when incrementally increasing the number of included ecoregions, which were continuously prioritized by species richness (blue), endemism (red), ∂-endemism (black), and threat (Red List categories CR+EN; green). Grey lines denote mean, 5% and 95%confidence limits when ecoregions are randomly selected (10000 simulations). Symbols represent species in ecoregions selected by the Global200 (circles) and Conservation International's Hotspots (diamonds; larger x-values for ecoregions within or overlapping Hotspots; smaller x-values for ecoregions within Hotspots).

All assessed prioritization schemes perform well in the accumulation of threatened species compared to random ecoregion selection, but major differences emerge between schemes. The number of species of any threat class (response measure; Figures 1A for AZE, 1B for EDGE, 1C for critically endangered and1D for endangered species, respectively) rises more rapidly when including increasing numbers of high-priority ecoregions using endemism and threat as prioritization metrics than for ∂-endemism and richness. The number of accumulated species exceeded the numbers expected by random ecoregion selection for richness, endemism and threat, even for the highest-ranking ecoregions. This applied to ∂ -endemism only for AZE (Figure 1A), whereas it required 100 ecoregions for EDGE (Figure 1B), 71 for CR (Figure 1C) and 69 for EN (Figure 1D) to exceed random ecoregion selection. In general, ∂ -endemism accumulated species slower than richness for the top ranks but outperformed richness after accumulating 81 to 242 ecoregions (AZE and EDGE species in Figures 1A and 1B, respectively). Both endemism and ∂ -endemism reached asymptotes at around 400 ecoregions, whereas richness arrived at that level only after all ecoregions were included. Accumulated values for Hotspots and Global200 were similar for EDGE and EN (Figure 1B, D), but Hotspots outperformed Global200 for AZE and CR (Figure 1A, C). Endemism and ∂ -endemism schemes were better than the Red List-based scheme once 200 to 300 ecoregions accumulated for all target threat categories, except EDGE. All schemes performed similarly for the top 100 ecoregions (an arbitrary cut-off, [26]) in capturing target species (Table S1).

**Figure 2:**
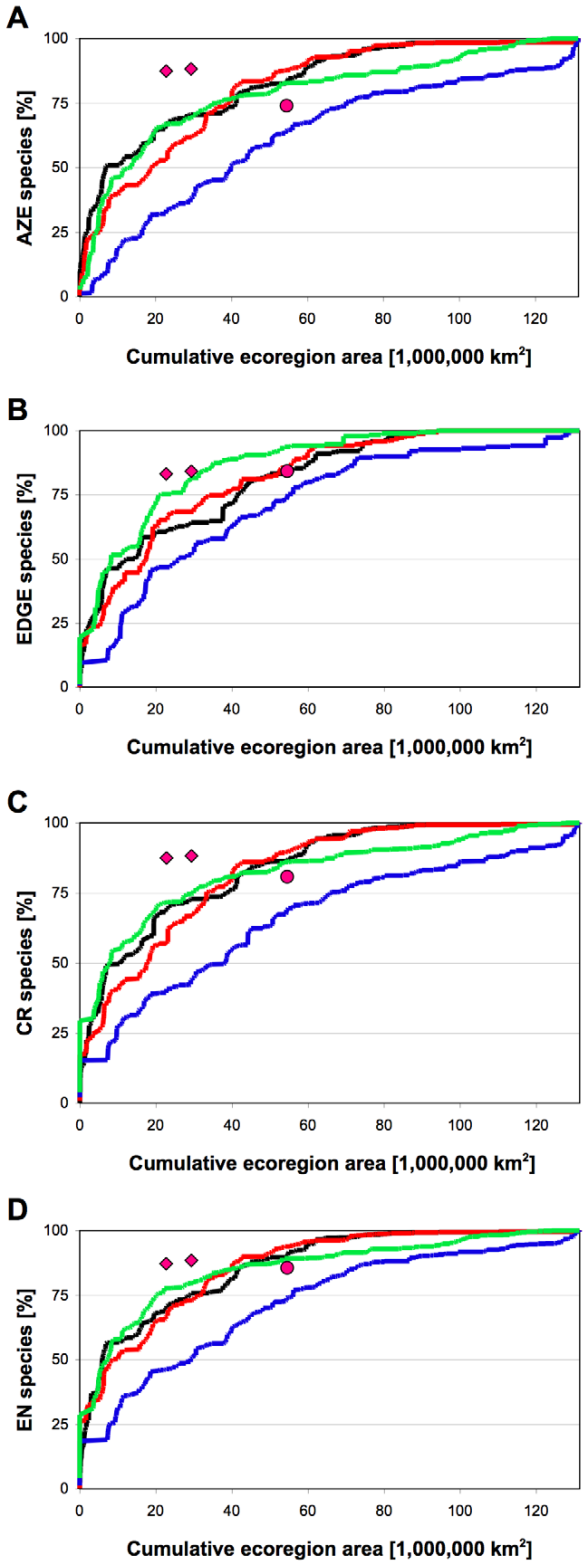
Accumulation of species over prioritized ecoregion area. Target species are EDGE (A), AZE (B), Red List's critically endangered CR (C) and Red List's endangered EN (D) species. Lines and symbols as in Figure 1.

We also evaluated the accumulation of target species over area (Figure 2) because of the known correlation between ecoregion size and species richness [10], and the observed biases of top-ranking ecoregions towards large size for richness and small size for ∂-endemism (Table S1, Figure S1). Considerable differences between the rank-based metrics emerge when 10% of area [10] is selected. Whilst richness always accumulated slowest, ∂-endemism and endemism amassed species equally and gathered target species comparable to, or often better, than the Red List-based prioritization scheme (Figure 2). Hotspots performed remarkably well for all threat classes, partly explicable by the inclusion of all major island systems in the newest iteration of the Hotspots [32].

**Figure 3:**
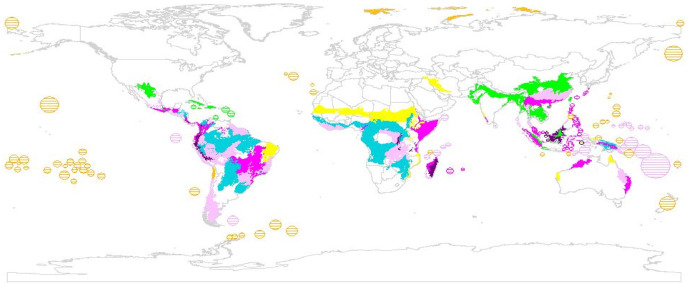
World map of ecoregions ranked according to species richness, endemism, ∂-endemism and threat. The congruence of the 100 highest ranking ecoregions of each prioritization scheme is shown (endemism only: yellow, ∂-endemism only: orange; richness only: turquoise; two, three or all metrics: light, medium or dark purple, respectively). Small islands, which are too small to be seen on the map, are highlighted by hatching.

By selecting the top 100 ranked ecoregions identified by the different schemes, we generated a new conservation priorities world map for land vertebrates containing 243 ecoregions (Figure 3). It shows that only nine ecoregions are shared between schemes and 136 (56%) are unique to each (richness: 16.5%; endemism: 4.1%; ∂-endemism: 17.3%; threat: 18.1%). 88 (36%) were outside the Global200 and 75 (31%) outside Hotspots. Species selected by ∂-endemism are underrepresented by alternative schemes (Figure S2).

**Figure 4:**
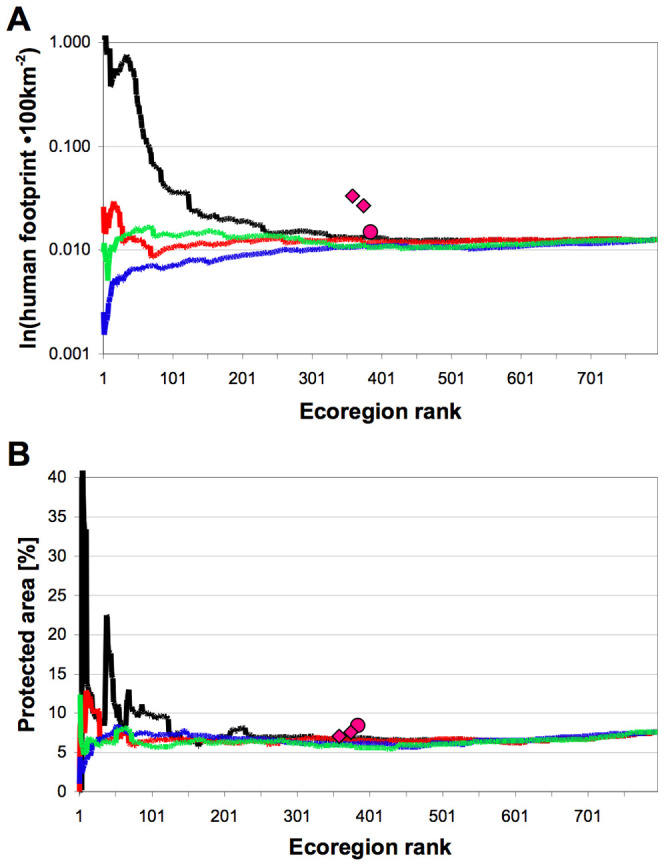
Average human footprint (A) and the percentage of protected area (B) over prioritized ecoregion rank. Lines represent values when incrementally increasing the number of included ecoregions, which were continuously prioritized by species richness (blue), endemism (red), ∂-endemism (black), and threat (Red List categories CR+EN; green). Symbols represent ecoregions selected by the Global200 (circles) and Conservation International's Hotspots (diamonds; larger x-values for ecoregions within or overlapping Hotspots; smaller x-values for ecoregions within Hotspots). Human footprint data and protected area sizes are from the WWF databases. The human footprint refers to the average footprint index, ranging from 0 to 100, and is scaled here to 100 square kilometres.

Finally, we analyzed protected area coverage and ‘human footprint’ [33] of prioritized ecoregions for richness, endemism and threat as a surrogate of their conservation status. The human footprint maps the continuum of human influence over terrestrial area and is similar to the ‘ecological footprint’, which estimates the amount of area necessary to support the consumption of one or more persons [33]. Richness-prioritized ecoregions were least impacted by current anthropogenic activities, but had lower protected area coverage (Figure 4). In contrast, human footprint and extent of protected areas were highest in ∂-endemism-prioritized ecoregions. Threat-prioritized ecoregions are relatively high for human footprint and poor in protected areas. Values for endemism-prioritized ecoregions fell between richness and ∂-endemism. As expected, hotspots appear as areas with very high human footprint. These results confirm the need for very distinct conservation strategies in line with the different prioritization metrics available. Within richness-prioritized areas, not enough land is currently protected. This contrasts with the smaller, more endemic areas where both nominal protection and human impacts are highest. Ecoregions with high species richness are large continental areas, so the efficient allocation of conservation resources will be better achieved by wiser political decisions related to agricultural expansion and natural resources exploitation, alongside the more traditional purchase and creation of protected areas [34]. Although the human footprint in these areas is still relatively low, projected land-use change is high [3]. The higher anthropogenic pressures in ecoregions prioritized by ∂-endemism and, to a lesser extent, those prioritized by endemism, islands and highlands in particular, make further extinctions imminent [26], [35]. This is because even when the proportion of protected area is high, in islands for example, these areas may still be under severe human pressure (and especially threatened by invasions of animal and plant). An example is the Galápagos Islands, which UNESCO declared in 2007 as “Worldwide Heritage in Danger” because of the imminent threats from invasives and tourism despite its protected status [36]. Although here the protected areas network is relatively well developed, significant gaps exist [35], which also need to be urgently addressed, especially as the impact of climate change will be particularly strong [3].

Strategic priority setting, if implemented, can support effective conservation action [37]. This has been achieved with great success, especially in Hotspots [32]. However, our analyses have shown that alternative prioritization approaches, each with different assumptions, are needed in targeting endangerment since they choose different sets of species (Figure S2) and priority areas (Figure 3); hotspots of richness, threat and endemism schemes vary greatly in their utility for conservation [17]. The development of a multitude of divergent sets of priority areas, often competing rather than complementary, is problematic because it creates redundancies in planning and conservation action by different organizations and distracts from a “commonly adopted blueprint for action” [12].

The choice of any particular metric is in some sense arbitrary, largely influenced by judgments rooted in the underlying conservation philosophy. Adoption of one single scheme also divides the conservation community. We therefore propose that our new global priority map, which combines highest-ranking ecoregions identified by several significant and complementary metrics is used. The map also permits the selection of ecoregions that are otherwise missed by some schemes (e.g. small islands). This allows worldwide conservation organizations to select areas to suit each organization's strength and philosophy. This, without doubt, should foster complementarity between organizations, thus reducing economic redundancies and increasing co-operation to protect biodiversity in the most efficient manner [38], [39]. By doing this, we reiterate what others have argued before [20], [40], it is mistaken to assume that there should be one common priority-setting method, “blue print” or “silver bullet”. Fixating on a single approach to biodiversity conservation increases the risk that alternative ways to prioritize ecoregions for conservation fall off-radar.

## Materials and Methods

### Data Sources

We used 796 out of the total of 867 WWF terrestrial ecoregions, excluding data deficient mangrove and some arctic ecoregions [10], [26]. The associated WWF “Wildfinder” database (Table S2 [28]) lists the presence/absence per ecoregion and their 2006 IUCN Red List [27] status of the world's terrestrial amphibians (*n* = 4797), reptiles (*n* = 7483), birds (*n* = 9470) and mammals (*n* = 4702), excluding introduced species, commensals, vagrants or passage migrants [10].

We corrected inconsistencies between WWF “*Wildfinder*” database, AZE and EDGE species (Table S2) stemming from different species and genera names, and use of synonyms. We identified ecoregions of occurrence for the 144 AZE and EDGE species that were not listed in *Wildfinder* and 62 species that were listed but had no ecoregions assigned. As all species in question were endemic, we could use the AZE [12] and EDGE [21] databases' point estimates of each distribution to assign ecoregions using ArcView GIS 3.3 (Environmental Systems Research Institute Inc) and the Point Stat Calc Extension [41]. We focussed on the most endangered taxa, namely the Red List's threat categories CR and EN. Of these, a total of 103 species had no ecoregions assigned in *Wildfinder*. As these represented a relatively small proportion of the overall data set of 1973 endangered and critically endangered, they were excluded as data-deficient species (see also [42], where 208 of 700 threatened anurans in the Neotropics were excluded). Ten EDGE species and 11 AZE species were excluded as these were non-terrestrial, or occurred outside the 796 ecoregions. In total, we used 740 AZE species and 190 EDGE species.

### Continuous Prioritization Metrics

Establishing “priority areas” for conservation relies on the representation of patterns and/or processes of biodiversity. The aim of detecting such “hotspots”, *sensu lato*, is to maximize protection from threats to the persistence of important areas of global species richness or endemism [43]. The most commonly used metrics to identify and prioritize important conservation areas for biodiversity are endemism and endangerment [7], [18], [10]. Following this approach, we prioritized ecoregions by ranking them according absolute endemism and threat. We compared the results directly with species richness. After database amendment and correction, we calculated absolute numbers for all species, endemic species and highly threatened species (Red List's threat categories CR and EN) in each ecoregion.

Ties were resolved by ecoregion size for species richness (the smaller the size the higher the rank). A total of 277 ecoregions had no endemic species and 17 ecoregions no threatened species. Resolving ranks by ranked richness for endemism and threat, which we adapted, reflects the importance of these ecoregions for biodiversity better than resolving ranks by ecoregion size. However, the manner ties are resolved have no impact for high-ranking ecoregions here as these ecoregions without endemic or threatened species are always of low priority for their respective metrics.

After ranking according to absolute species numbers, we also prioritized using taxonomically standardized values across the four taxonomic classes to avoid a single class overwhelming the others [10], [26]:
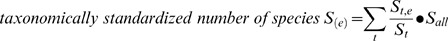
where *t* is the taxonomic class, *S _t,e_* is the number of species in each taxonomic class for each ecoregion, *S _t_* is the number of species in each taxonomic class across all ecoregions, *S _all_* is the number of species across all taxonomic classes and ecoregions (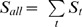), and *S_(e)_* refers to either all species, endemic species or threatened species within ecoregions *e.* Absolute and taxonomically standardized species numbers and the resulting ranks were highly correlated for richness (Pearson's *r* = 0.95 for species number, Spearman's rank correlation *r_S_* = 0.96 for ranks), endemism (*r* = 0.98 and *r_S_* = 0.98, respectively) and threat (*r* = 0.97 and *r_S_* = 0.96, respectively). Mean, median and ±SD rank differences were 0.0, 2.0 and ±64.1 for richness, 0.0, 2.0 and ±40.1 for endemism and 0.0, 4.5 and ±61.2 for threat, respectively. Because correlations between estimators are all high (95% to 98%) we focus on taxonomically standardized values - henceforth denoted richness, endemism and threat - to assure consistency with prior studies [10], [26].

A gap analysis of global protected-area network [35] has identified small tropical islands as the most serious absences from the map of priorities for expanding the global network. Small islands are typically missed by richness, absolute endemism or threat as they mostly exhibit low species richness. Therefore, they tend to have relatively low absolute numbers of endemic and threatened species despite that these represent high proportions of their species numbers. Elsewhere, we have shown that empirical logit transformation of endemism, which constitutes an adjustment of absolute endemism with richness, ranks high the ecoregions that are relatively rich in endemics, but poor in species [26]. We used the empirical logit function and not proportional endemism because Principle Component Analysis, PCA, identified it as best suited to characterise patterns of endemism across the terrestrial ecoregions [26], [44]. The empirical logit transformation for each ecoregion *e* can be rewritten as:
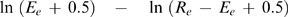
where *R_e_* is the taxonomically standardized number of species, *E_e_* is the taxonomically standardized number of endemics and the term “+0.5” is an adjustment to avoid difficulties when *E_e_* = 0. We thus refer to it as ∂-endemism. Ecoregions that are relatively rich in endemics, but poor in species, are typically higher ranked by ∂-endemism as by other measures [26] as exemplified by the Galápagos Islands “scrublands mosaic” ecoregion, ranked 5^th^ by ∂-endemism, 31^st^ by endemism, 708^ th^ by richness and 222^ th^ by threat.

### Categorical Prioritization Schemes

We also evaluated the two most widespread categorical prioritization schemes used for selection of areas for global conservation: the newest version of CI's Hotspot approach [32] and WWF's Global200 ecoregions [25]. The newest version of CI's Hotspot approach [10] follows ecoregion boundaries closely. We identified ecoregions totally or partially within hotspots by manual comparison of GIS shapefiles of ecoregions and all core and extended hotspots. Of the 796 ecoregions used in this study, hotspots contained 355 and 16 ecoregions fully or partially. We conducted Hotspot analyses under two scenarios, one which included only those ecoregions that are totally within Hotspots, and another which incorporated all ecoregions whether fully or partially overlapping with Hotspots. The Global200 approach is based on ecoregions and is included in the WWF ecoregions database. Many are composed of multiple ecoregions (a total of 384 of the 796 ecoregions we used constitute the Global200).

### Prioritization Efficiency

We assessed how such continuous and categorical prioritization schemes perform in the accumulation of species of special interest (e.g., threatened taxa). We used all listed AZE mammals, birds, reptiles and amphibians [30], top 100 EDGE mammals and top 100 EDGE amphibians [31; only mammals and amphibians have received EDGE scores so far], as well as the Red List categories CR and EN. We calculated the number of target species for increasing numbers of prioritized ecoregions. Non-endemic species, i.e. species that occurred in more than one ecoregion, were considered only at the first (highest-priority) ecoregion where the species occurred. To determine whether the percentages of target species captured by prioritization were significantly different than by chance alone, we simulated 10,000 random ranking schemes by randomization using a random number generator (computer program written in C). A previous study aimed at identifying and characterizing biological patterns underpinning the geographic distribution of richness and the correlation with endemism used geographically restricted randomization by constraining randomizations by both biome and realm [10]. Here, we evaluated possible prioritization by conservation managers and institutional policies, which may or may not consider geographical associations. We thus employed unrestricted randomizations. We then calculated accumulated target species for increasing numbers of prioritized ecoregions, and calculated the mean and 5% and 95% confidence intervals.

### Characteristics of Prioritized Ecoregions

As a measure of human influence within each of the Earth's ecoregions we used the ‘human footprint’ *sensu* [33], which used four types of data as proxies for human influence: population density, land transformation, accessibility, and electrical power infrastructure. The human footprint data are continuously mapped over terrestrial space [33]. The WWF databases [21] include a summary statistics in the form of minimum, maximum, average and summary footprint indices per each ecoregion. We plotted the accumulation of these indices over prioritized ecoregions ranks. As all plots revealed essentially the same trend, we used the average footprint index per ecoregions. As a proxy for conservation status we used the percentage land area within each ecoregion legally dedicated to the protection and maintenance of biological diversity, encompassing a number of IUCN management categories.

We mapped the top 100 ranked ecoregions identified by each of the four continuous prioritization schemes to visualize intersection of schemes and non-congruency.

## Supporting Information

Figure S1 Accumulation of area size when selecting ecoregions by different prioritization methods. Lines represent the cumulative area size of ecoregions when incrementally increasing the number of included ecoregions, which were continuously prioritized by species richness (blue), endemism (red), δ-endemism (black) and threat (green). Symbols denote the area size of ecoregions selected by the Global200 (circles) and Hotspots (diamonds; larger x-values: all ecoregions within or overlapping Hotspots; smaller x-values: ecoregions within hotspots only).
(0.16 MB TIF)

Figure S2 Number of species unique to the prioritization method over prioritized ecoregion ranks. Lines represent unique species when incrementally increasing the number of included ecoregions, which were prioritized on the basis of species richness (blue), endemism (red), δ-endemism (black) and threat (green). Only the 250 highest ranking ecoregions are shown. Target criteria include EDGE (A), AZE (B), Red List's critically endangered CR (C) and Red List's endangered EN (D) species.
(0.47 MB TIF)

Table S1 Number of endangered species captured by prioritization metrics.
(0.04 MB DOC)

Table S2 Databases used.
(0.04 MB DOC)
