# Supplementary figures and images for: Ecoregion Prioritization Suggests an Armoury Not a Silver Bullet for Conservation Planning
Source: PLoS One. 2010 Jan 27;5(1):e8923. doi: 10.1371/journal.pone.0008923 (PMC2811746; doi:10.1371/journal.pone.0008923)

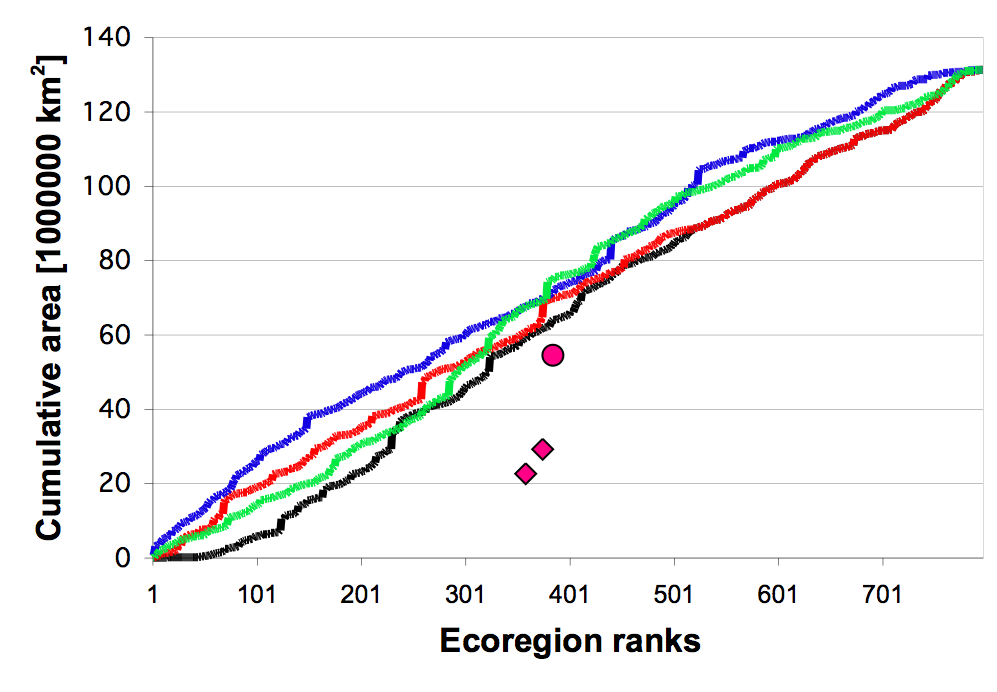

Supplement: Figure S1 — Accumulation of area size when selecting ecoregions by different prioritization methods. Lines represent the cumulative area size of ecoregions when incrementally increasing the number of included ecoregions, which were continuously prioritized by species richness (blue), endemism (red), δ-endemism (black) and threat (green). Symbols denote the area size of ecoregions selected by the Global200 (circles) and Hotspots (diamonds; larger x-values: all ecoregions within or overlapping Hotspots; smaller x-values: ecoregions within hotspots only). (0.16 MB TIF) [file pone.0008923.s001.tif]

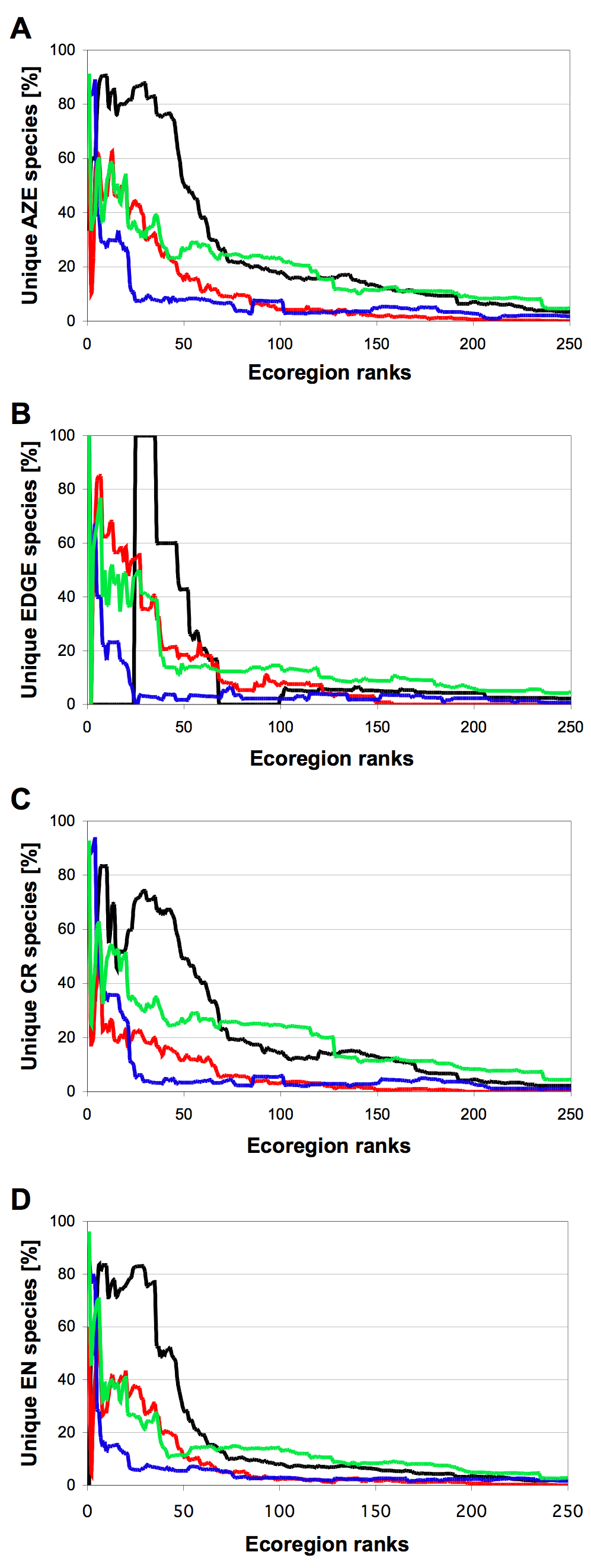

Supplement: Figure S2 — Number of species unique to the prioritization method over prioritized ecoregion ranks. Lines represent unique species when incrementally increasing the number of included ecoregions, which were prioritized on the basis of species richness (blue), endemism (red), δ-endemism (black) and threat (green). Only the 250 highest ranking ecoregions are shown. Target criteria include EDGE (A), AZE (B), Red List's critically endangered CR (C) and Red List's endangered EN (D) species. (0.47 MB TIF) [file pone.0008923.s002.tif]
