# Supplementary material for: Ecoregion Prioritization Suggests an Armoury Not a Silver Bullet for Conservation Planning
Source: PLoS One. 2010 Jan 27;5(1):e8923. doi: 10.1371/journal.pone.0008923 (PMC2811746; doi:10.1371/journal.pone.0008923)
Supplement: Table S1 — Number of endangered species captured by prioritization metrics. (0.04 MB DOC) [file pone.0008923.s003.doc]

Table S1. Number of endangered species captured by prioritization metrics.

| Scheme | Richness | Endemism | ∂-endemism | Threat | 95% CI |
| --- | --- | --- | --- | --- | --- |
| *Species captured by the top 100 ranked ecoregions* | | | | |  |
| CR (n) | 276 (42%) | 359 (54%) | 252 (38%) | 386 (61%) | 97-181 (15%-27%) |
| EN (n) | 578 (48%) | 753 (62%) | 558 (46%) | 775 (64%) | 248-378 (20-31%) |
| AZE (n) | 264 (36%) | 368 (50%) | 308 (42%) | 385 (52%) | 75-160 (10%-22%) |
| EDGE (n) | 94 (49%) | 108 (57%) | 64 (34%) | 104 (55%) | 35-64 (18%-34%) |
| Area (km^2^) | 25,896,331 (20%) | 18,996,737 (14%) | 5,721,602 (4%) | 14,213,541 (11%) |  |
| *Species captured by the top ecoregions representing 10% of total area* | | | | |  |
| CR (n) | 208 (32%) | 294 (44%) | 350 (53%) | 381 (58%) |  |
| EN (n) | 441 (37%) | 653 (54%) | 710 (59%) | 754 (62%) |  |
| AZE (n) | 166 (22%) | 320 (43%) | 402 (54%) | 369 (50%) |  |
| EDGE (n) | 59 (31%) | 85 (45%) | 95 (50%) | 103 (54%) |  |
| Ecoregions (n) | 51 | 69 | 140 | 94 |  |

Shown are the accumulated numbers for the top 100 ranked ecoregions and the highest ranking ecoregions that represent 10% of the total ecoregion size. Endangerment schemes are Red List CR, Red List EN, AZE species and EDGE species. Values in parentheses are percentages relative to the total numbers of species in each endangerment scheme for all 796 terrestrial ecoregions. 95% confidence intervals are included for the top 100 ecoregions.

## 
