# Supplementary material for: Ecoregion Prioritization Suggests an Armoury Not a Silver Bullet for Conservation Planning
Source: PLoS One. 2010 Jan 27;5(1):e8923. doi: 10.1371/journal.pone.0008923 (PMC2811746; doi:10.1371/journal.pone.0008923)
Supplement: Table S2 — Databases used. (0.04 MB DOC) [file pone.0008923.s004.doc]

Table S2. Databases used.

| Author | Database | Date accessed |
| --- | --- | --- |
| WWF | WildFinder Database (<http://www.worldwildlife.org/science/data/item1873.html>,  <http://www.worldwildlife.org/wildfinder/>) | 4 Aug 2008 |
| WWF | Terrestrial Ecoregions Base Global Dataset (<http://www.worldwildlife.org/science/data/item1874.html>) | 4 Aug 2008 |
| WWF | Terrestrial Ecoregions GIS Database (<http://www.worldwildlife.org/science/data/item1875.html>) | 4 Aug 2008 |
| WWF | Global 200 Ecoregions Database (<http://www.worldwildlife.org/science/data/item1878.html>) | 4 Aug 2008 |
| CI | Maps & GIS data (<http://www.biodiversityhotspots.org/xp/hotspots/resources/Pages/maps.aspx>) | 1 May 2009 |
| AZE | Species lists (<http://www.zeroextinction.org/>; M. Hoffmann, Conservation International, pers. communcation) | 16 April 2007 |
| EDGE | Species lists (www.edgeofexistence.org/; C. Waterman, ZSL, pers. communication) | 17 June 2008 |

## 
